# RADICAL study: rapid diagnosis of suspected bloodstream infections from direct blood testing using PCR/ESI-MS

**DOI:** 10.1186/cc14064

**Published:** 2014-12-03

**Authors:** D Brealey, N Libert, J Pugin, M O'Dwyer, K Zacharowski, M Mikaszewska-Sokolewicz, MP Maureau, DJ Ecker, R Sampath, M Singer, J-L Vincent

**Affiliations:** 1 University College Hospital, London, UK; 2 Military Hospital du Val-de-Grâce, Paris, France; 3 Hôpitaux Universitaires de Genève, Geneva, Switzerland; 4 The Royal London Hospital Barts, London, UK; 5 Universitätsklinikum Frankfurt, Germany; 6 Child of Christ Hospital, Warsaw, Poland; 7 Ibis Biosciences, Abbott, Carlsbad, CA, USA; 8 Erasme University Hospital, Université Libre de Bruxelles, Belgium, Belgium

## Introduction

Survival from sepsis and bloodstream infections (BSI) often depends upon rapid identification of the infecting pathogen and expeditious antimicrobial therapy. We report findings from the final analysis of RADICAL, a multicenter observational study that compared results from direct blood specimen testing using PCR/ESI-MS to standard microbiology in critically ill patients.

## Methods

Eight ICUs in six European countries participated. Patients with suspected infection plus ≥2 new onset SIRS criteria were enrolled and had an extra blood specimen taken for PCR/ESI-MS direct analysis. Results were compared to standard of care microbiology. All patients were followed up to 28 days or until inpatient death or discharge. A panel of three independent physicians reviewed a summary of each case to determine the potential impact upon patient management if results had been available for decision-making.

## Results

A total of 609 direct blood specimens from 543 patients meeting inclusion criteria were tested. Patient demographics and organ failure criteria observed were consistent with previously published sepsis studies. Culture/PCR comparisons were as follows: +/+ 54; +/- 13; -/ + 169; and -/- 393, respectively, for a sensitivity of 81%, specificity of 69%, PPV of 24% and NPV of 97%. The distribution of the organisms in the culture-/PCR+ group was similar to the culture+/PCR+ group and was reproducible in replicate testing with an independent blood draw. Analysis of additional sample types (lower respiratory tract or sterile fluids and tissues) corroborated direct blood findings in 58% of cases where multiple specimens from the same patients were analyzed. The independent expert panel would have considered a different course of care in 57% of the cases when PCR/ESI-MS was positive, 41% of which would have resulted in altered, instituted, or ceased antibiotic therapy, earlier.

## Conclusion

These results suggest that PCR/ESI-MS accurately detects the infecting pathogen in critically ill patients with BSI, and that its use might often lead to a different treatment. The test's ability to rule in or rule out infection approximately within 8 hours of the blood draw has added potential clinical and economic benefit, as it might minimize unnecessary use of antibiotics.

